# Individual dislocation identification in dark-field X-ray microscopy

**DOI:** 10.1107/S1600576725002614

**Published:** 2025-05-02

**Authors:** Sina Borgi, Grethe Winther, Henning Friis Poulsen

**Affiliations:** a Department of Physics Technical University of Denmark 2800Kongens Lyngby Denmark; b Department of Civil and Mechanical Engineering Technical University of Denmark 2800Kongens Lyngby Denmark; Ecole National Supérieure des Mines, Saint-Etienne, France

**Keywords:** diffraction microstructure imaging, X-ray imaging, X-ray microscopy, geometrical optics, dislocation identification

## Abstract

Dark-field X-ray microscopy (DFXM) has recently been introduced for 3D mapping of dislocations and their strain fields in bulk samples and with high angular resolution (10^−4^°). In this work, we investigate the minimum information needed to identify the type of an isolated dislocation, parameterized by its Burgers vector, line direction and slip plane. Forward projections of DFXM weak-beam images are generated for a face-centred cubic symmetry using geometrical optics simulations with realistic noise levels. Cross correlating one DFXM image with similar images representing all possible combinations of dislocation types, we find that the cross-correlation values for all non-identical images are below 0.7, clearly demonstrating the feasibility of this method of identification. Experimental DFXM images of isolated dislocations are compared with forward-modelled ones. Complete identification is demonstrated, with the exception of the sign of the Burgers vector. The performance improvement obtained by acquiring data from a 3D volume is explored. This work verifies the use of geometrical optics to simulate DFXM weak-beam images and supports the interfacing of DFXM data with discrete dislocation dynamics simulations.

## Abstract 2

Experimental and simulated dark-field X-ray microscopy images of isolated dislocations in bulk single-crystal aluminium were combined to identify the Burgers vector, slip plane and line direction of the dislocations. Burgers vector identification missed only the sign, and the line direction was determined with an error of less than 10°, sufficient for most applications.

## 1. Introduction

Dislocations play a crucial role in the plastic deformation of metals, governing key mechanical properties such as strength, ductility and toughness. The motion of these line defects in the crystal structure on close-packed layers (slip planes) facilitates the layers sliding over each other, enabling metals to undergo plastic deformation (Hirth & Lothe, 1992 ▸). With an increasing number of dislocations they form patterns, leading to a hierarchically organized structure which may comprise near-dislocation-free cells, bands and grains. To model the evolution of the entire microstructure, multiscale models are applied, *e.g.* involving discrete dislocation dynamics (DDD) (Devincre *et al.*, 2011 ▸; Po *et al.*, 2014 ▸; Sills *et al.*, 2016 ▸; Pachaury *et al.*, 2022 ▸) and continuum dislocation dynamics (CDD) (Mohamed *et al.*, 2015 ▸) simulations. Understanding the organization of dislocations and their evolution is essential for predicting material behaviour under stress in metals and alloys, but it also governs the mechanical properties of other crystalline materials, *e.g.* fracture toughness and thermal stability in ceramics (Porz *et al.*, 2021 ▸).

Identifying and characterizing individual dislocations – in terms of Burgers vector and line directions – thus provides valuable insight that can inform the design of stronger and more resilient materials.

Traditionally, advanced electron microscopy techniques are used to visualize dislocations by revealing defects in the crystalline structure. Transmission electron microscopy (TEM) enables direct observation of the individual dislocation lines with (near) atomic-scale resolution (Williams & Carter, 2009 ▸). Electron backscatter diffraction (EBSD) typically provides insights into collective dislocation structures and grain–boundary interactions (Moussa *et al.*, 2017 ▸). Recently, high-resolution EBSD (HR-EBSD) has been able to reveal individual dislocations (Ernould *et al.*, 2022 ▸). However, all electron-based techniques are limited to visualizing only the surface or thin slices of samples, making it difficult to acquire 3D maps of the microstructure on all relevant length scales and to study the dynamics under bulk conditions.

Of particular relevance for low-dislocation-density systems such as semiconductor devices, the local distortion of the crystal structure caused by dislocations can also be probed by X-ray topography (Lang, 1997 ▸; Ohler *et al.*, 1997 ▸). Three-dimensional information may be gathered *e.g.* in Laue geometry by tomographic reconstruction (Ludwig *et al.*, 2001 ▸) or in Bragg configuration by combining section topography with focused sheet-shaped X-rays (Yoneyama *et al.*, 2023 ▸).

Mapping on a finer length scale is available via synchrotron-based X-ray diffraction. This has been explored by Bragg coherent diffraction imaging (BCDI) (Ulvestad *et al.*, 2017 ▸; Cherukara *et al.*, 2018 ▸) and differential aperture X-ray microscopy (Guo *et al.*, 2015 ▸; Guo *et al.*, 2020 ▸). In BCDI, the dislocations shift the phase of the scattered X-rays, allowing for the reconstruction of 3D displacement fields within a crystal at nanometre-scale resolution. However, BCDI is limited in volume to mapping out nanoscale crystals or small regions of larger crystals.

Recently, dark-field X-ray microscopy (DFXM) has emerged as a novel diffraction-based imaging technique capable of non-destructively mapping dislocations and their displacement fields in three dimensions within bulk crystalline materials (Jakobsen *et al.*, 2019 ▸; Simons *et al.*, 2019 ▸). DFXM uses high-energy incoherent X-rays combined with dark-field conditions to image specific crystallographic planes deep within the material (Simons *et al.*, 2015 ▸; Poulsen *et al.*, 2017 ▸; Poulsen *et al.*, 2021 ▸). With sub-micrometre resolution, DFXM can visualize both structures formed by multiple dislocations (Zelenika *et al.*, 2024 ▸) and individual dislocations in volumes as large as a cubic millimetre (Yildirim *et al.*, 2023 ▸). The technique exhibits superior angular resolution, allowing for mapping the mechanical field around the dislocations. DFXM has also been employed to capture movies of dislocation motion in real time, *e.g.* to study phase transitions in ferroelectrics (Simons *et al.*, 2018 ▸) and the influence of dislocations on melting (Dresselhaus-Marais *et al.*, 2021 ▸).

The aim of this paper is to provide a methodology for identifying the nature of the individual dislocations in DFXM images. We shall assume that the incident X-ray beam illuminates a layer in the sample. The dislocations are fully characterized by the magnitude and direction of their Burgers vectors and the direction of their dislocation lines. The slip plane on which a dislocation moves is spanned by these two directions.

There are several ways DFXM can visualize and quantify dislocations. First, as demonstrated in previous studies (Jakobsen *et al.*, 2019 ▸; Simons *et al.*, 2019 ▸; Brennan *et al.*, 2022 ▸), dislocations are directly visible in the raw detector images in the weak-beam condition, where the sample is tilted slightly with respect to the diffraction condition of the undistorted lattice.

Second, rocking curve imaging, where images are captured while varying the Bragg condition, can provide detailed information about the lattice distortion field surrounding a dislocation (Caliste *et al.*, 2021 ▸). In both cases, mapping may be done in two or three dimensions (by scanning the sample in the direction perpendicular to the incident line beam). The 3D mapping gives information on the glide planes and directions of the dislocations.

In this paper, we investigate the potential for classifying dislocations on the basis of a single weak-beam position. Rocking curve imaging comprises more information and will naturally lead to more robust and/or detailed classification schemes, but also takes longer in terms of data acquisition; this will be explored elsewhere. For simplicity we will consider only a face-centred cubic (f.c.c.) lattice, and we will specifically focus on pure aluminium. The study is performed with the use of numerical simulations (forward projections) of DFXM images. Subsequently, the tool derived is demonstrated for identification of dislocations in experimental DFXM data.

## 2. Methods

In this section we will present the methods used to identify individual dislocations, describe how comparisons between simulated images and experimental images have been made, and explain the quantitative correlation metrics used.

### 2.1. Experimental

**Figure 1:**
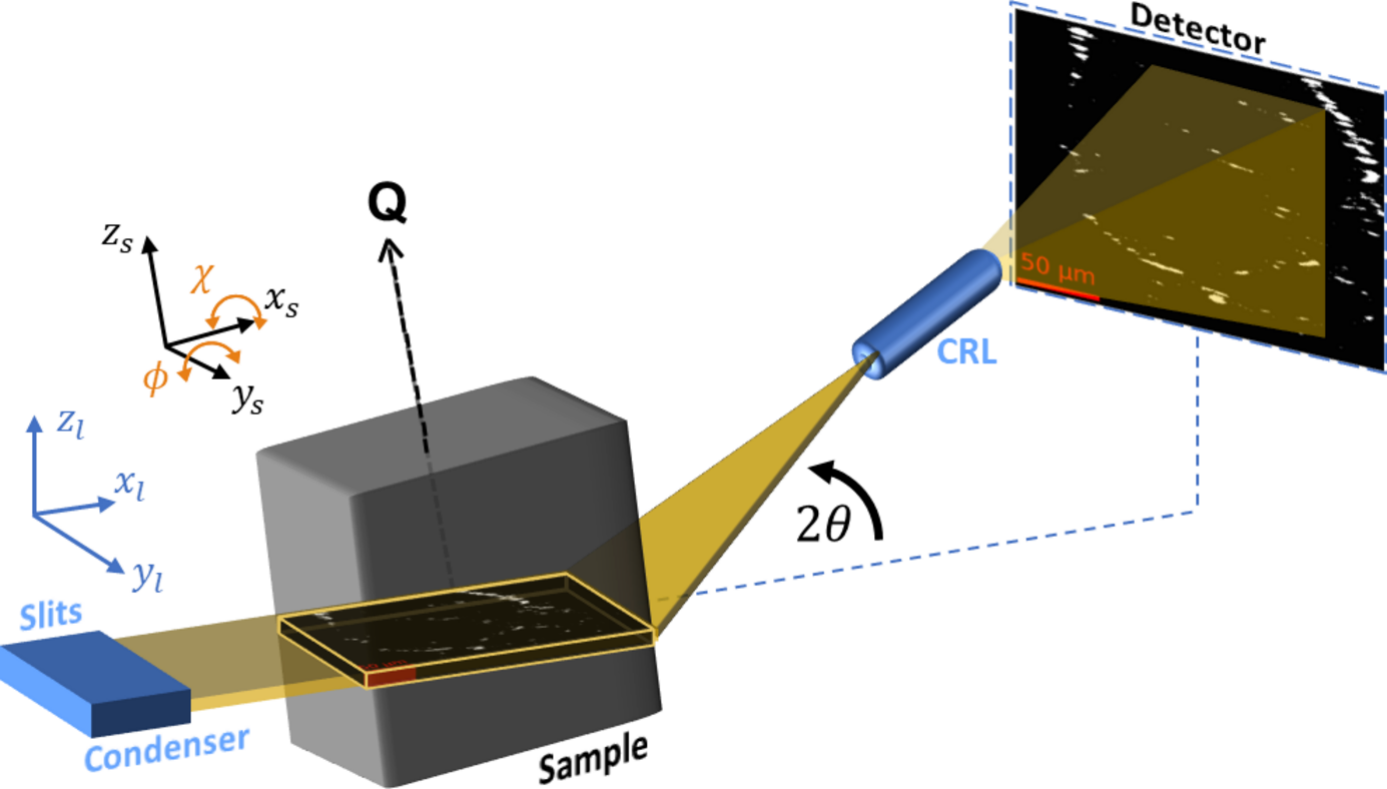
Schematic diagram illustrating the DFXM setup in both the laboratory (*x*_ℓ_, *y*_ℓ_, *z*_ℓ_) and sample (*x*_s_, *y*_s_, *z*_s_) coordinates. The incoming beam, aligned with the *x*_ℓ_ axis, is shaped by a 1D condenser to illuminate a specific (*x*_ℓ_, *y*_ℓ_) layer within the sample. The diffraction signal at the angle 2θ is focused by the CRL, creating a real-space image of the illuminated layer on the detector. The rotations χ and ϕ are shown around the *x*_s_ and *y*_s_ axes, respectively.

The experimental data were collected on the DFXM station of beamline ID06 at the European Synchrotron Radiation Facility (ESRF, Grenoble, France) (Kutsal *et al.*, 2019 ▸). The energy of the incoming beam was 17 keV with a bandwidth of Δ*E*/*E* = 10^−4^. The incoming beam is shaped to a line beam height of 0.6 µm (FWHM) and a width of 0.5 mm. As illustrated in Fig. 1 ▸, this line beam illuminates a plane deeply embedded in the bulk of the sample. Three-dimensional mapping is obtained by repeating measurements for a set of planes at equidistant positions in *z*_ℓ_. The sample was a 1 mm thick well annealed aluminium single crystal deformed just beyond the yield point in tension. The tensile axis is chosen to be identical to the scattering vector direction 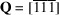, where **Q** denotes the direction of the scattering vector in reciprocal lattice units. The magnitude of the scattering vector is given by |**Q**| = (4π/λ)sin(θ), where λ is the incident wavelength and 2θ the scattering angle.

The diffracted signal in the vicinity of **Q** is imaged using a compound refractive lens (CRL) as an objective, producing a magnified real-space image of the plane. This image is subject to an affine transformation caused by the oblique viewing angle, given by 2θ. In the case presented below, the in-plane spatial resolution is about 200 nm (along *y*_ℓ_) × 615 nm (along *x*_ℓ_). The corresponding total field of view (FOV) is 432 × 1574 µm (in practice, the FOV in the *x*_ℓ_ direction is limited by the 1 mm thickness of the sample).

**Figure 2:**
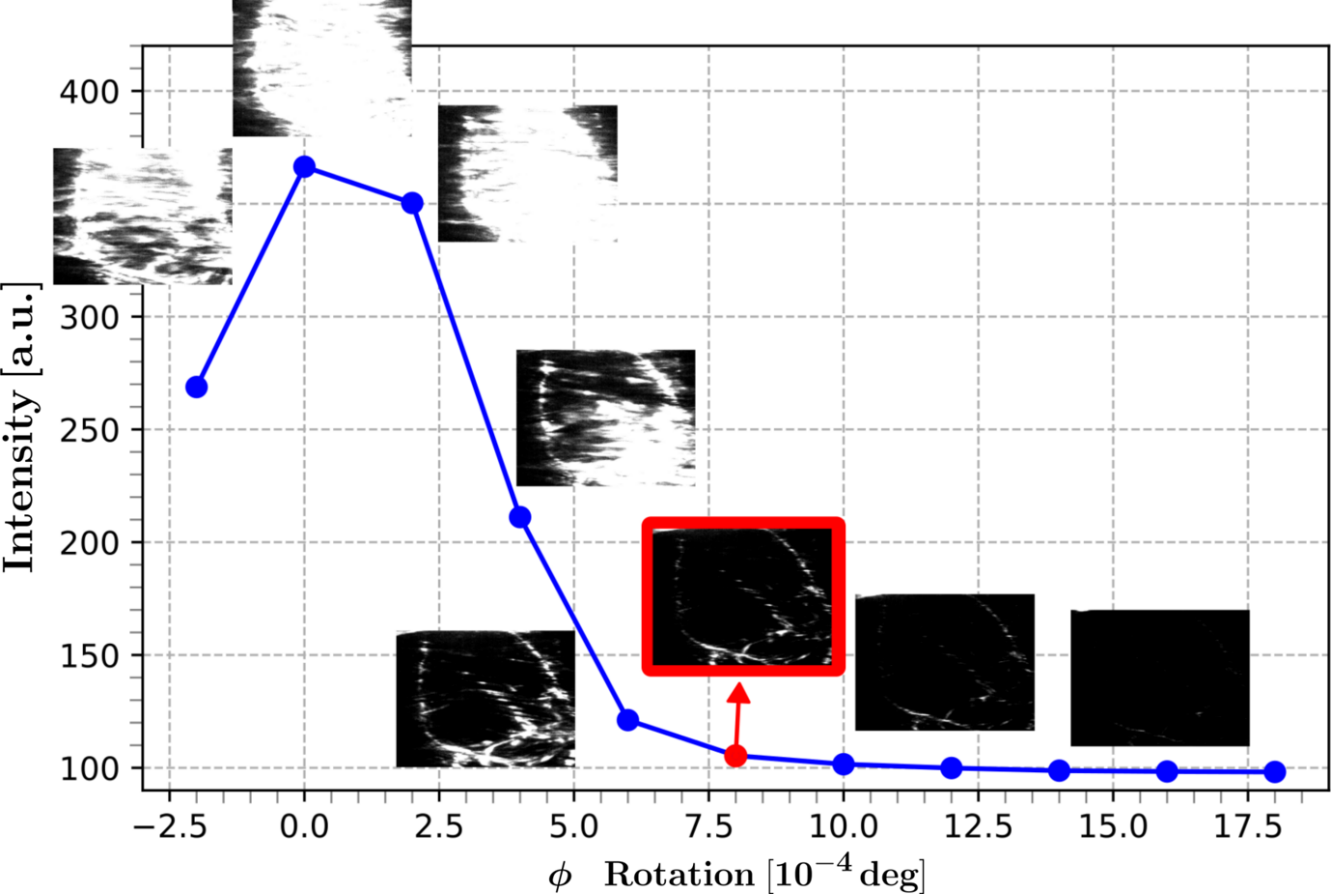
Example of experimental data: a rocking curve around the nominal value for the **Q** = 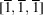 reflection (ϕ = 0) of the aluminium single crystal studied. As the sample is rotated away from ϕ = 0, the mean intensity of the acquired images (blue line) decreases. Insets of raw images are added at different points of the rocking curve, illustrating the strong-beam condition (crystal lattice in diffraction) and the weak-beam condition (dislocation distortion fields in diffraction), until the sample is completely rotated out of the diffraction condition. Most of the images used in this work were acquired at 8 × 10^−4^°, highlighted in red. The images in the insets have a FOV of 260 × 200 µm.

The sample is mounted on top of a goniometer, enabling tilting of the diffraction vector by angles ϕ and χ and translations of the sample in *x*_s_, *y*_s_ and *z*_ℓ_ (Fig. 1 ▸). This facilitates multiple scan modes that combine rotations and translations of the sample. For this experiment, we combined rocking curves with *z*_ℓ_ axis translations. In the rocking curve scan, images are acquired while stepping the ϕ rotation (that is, sample rotation around the *y*_s_ axis; Fig. 1 ▸) with a 10^−4^° sensitivity. Examples of the resulting raw images are provided as insets in Fig. 2 ▸. At ϕ = 0°, the undistorted part of the crystal diffracts (strong-beam condition). Small changes in ϕ bring the distorted crystal lattice around the dislocations into diffraction. By inspection of the image series of the rocking curve (Fig. 2 ▸), we assess that the distortion field gives the best (weak-beam) contrast to the undistorted crystal structure at ϕ = 8 × 10^−4^° (highlighted in red).

### 2.2. Geometrical optics simulations

The simulated images in this work were all created with a forward model based on geometrical optics (GO). It assumes kinematic scattering, and as such is only relevant for weak-beam conditions. The formalism is described by Poulsen *et al.* (2021 ▸) ▸, where it was implemented as a MATLAB script. The code was optimized by Borgi *et al.* (2024 ▸) ▸ as a Python script.

Briefly, GO derives scattering probabilities as a convolution in six dimensions (reciprocal and direct spaces) between an instrumental resolution function and the position and wavevector transfer of a diffraction event. The model describes how the intensity of the scattered X-rays is attenuated as they pass through the sample and the objective, and how they form an image on the detector. In reciprocal space a Monte Carlo approach is exploited in which ray-tracing simulations are confined by the properties of the incoming beam, the objective in the diffraction path and the detector. The direct-space component of the resolution function accounts for the effects of the sample’s spatial configuration, such as the shape and size of the scattering objects, the sample’s position relative to the incoming beam and the geometry of the detector.

The GO formalism paper by Poulsen *et al.* (2021 ▸) ▸ also introduces a description of the different coordinate systems to define the various parameters of the simulations:

(i) *The laboratory coordinate system*, subscript ℓ, with the incoming beam parallel to the *x* axis, as seen in Fig. 1 ▸.

(ii) *The sample coordinate system*, subscript s, in general defined by axes relevant to the sample geometry or manufacturing. In our case this is defined by a rotation by θ around the *y*_ℓ_ axis.

(iii) *The grain coordinate system*, subscript g, normally defined by the orientation of a particular grain. Here it is used to rotate dislocation systems (see below).

(iv) *The dislocation coordinate system*, subscript d, based on the Burgers vector, slip plane and line direction of the individual dislocation.

Rotation matrices are used to convert between the different coordinate systems. Below we summarize the most relevant micromechanical expressions and coordinate transformations for the current work.

In GO, diffraction events are described in terms of a micro­mechanical model, based on the displacement gradient tensor field **F**(*x*, *y*, *z*). Specifically, we define the *dislocation coordinate system* in terms of one isolated and straight dis­location, with the dislocation line direction along the *z* axis. We have 
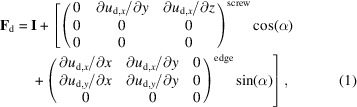
with **I** being the identity matrix and **u**_d_ the displacement field. The angle α between the Burgers vector and the dislocation line is 0 or 180° for pure screw segments and 90 or 270° for pure edge parts. Angles in between represent dislocations of mixed character.

Next, consider an arbitrary slip system defined by a Burgers vector **b**, a slip-plane normal **n** and a line direction **t**. We define the grain system to have **b**, **n** and **t** along the *x*_g_, *y*_g_ and *z*_g_ directions, respectively. Note that for this work the *grain coordinate system* spanned the whole sample, as the sample was defined as an aluminium single crystal with f.c.c. symmetry to match the experimental data. Hence, 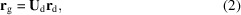

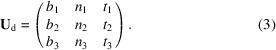


The sample system is partly defined by the sample being in the diffraction condition. The remaining degree of freedom is given by the ‘mounting’ of the sample. Here we specify that a certain crystallographic direction is parallel with the *y* axis of the laboratory coordinate system, *y*_ℓ_. Hence, 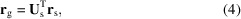

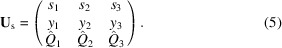
Here, 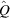 is the normalized scattering vector and **s** is the resulting crystallographic direction that is parallel with the *x*_s_ direction, given by 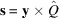.

As already mentioned, the sample system is related to the laboratory system by a rotation θ around *y*_ℓ_:
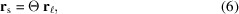

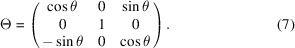


In total, the relation between the laboratory system and the dislocation system becomes 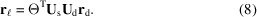


**Figure 3:**
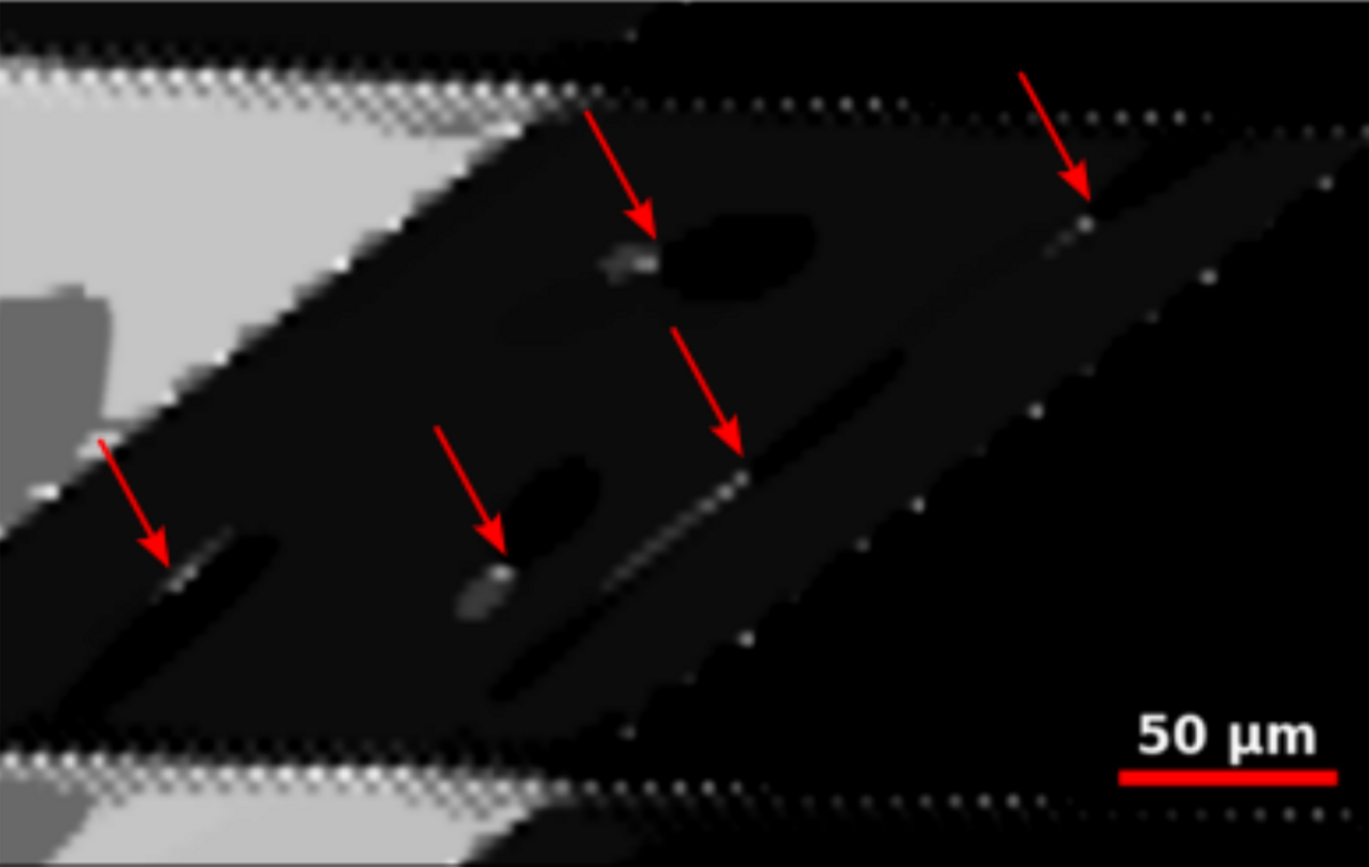
Simulated DFXM image of a layer in a single crystal of aluminium ([111] reflection), displaying the contrast of five random edge dislocations with different Burgers vectors (highlighted with arrows) within a domain.

To illustrate how dislocations look in the simulated DFXM weak-beam images, we show the forward projection of a phantom for a setting close to the [111] reflection in Fig. 3 ▸. The phantom comprises walls of edge dislocations which delineate a domain. Within this domain five edge dislocations of random Burgers vectors (highlighted with red arrows) are located. Each of these exhibits its own distortion field.

### 2.3. Cross-correlation metric

To determine the quality and uniqueness of the dislocation type identification, we will rely on cross correlation either between a set of simulated images or between an experimental image and a simulated image. The cross correlation between such a set of images is determined using fast Fourier transforms (FFTs). When comparing simulated weak-beam images with experimental ones, an additional step was introduced as described below.

Before the cross correlation was calculated, the images were normalized. Three normalization methods were compared (see the supporting information). From histograms of the corresponding cross-correlation values for a test data set (Fig. S1 in the supporting information), the best normalization method was found to be *Z*-score normalization (Wittwer & Seita, 2022 ▸). In this we have for each pixel in each image *I*(*x*, *y*), 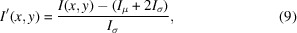
with *I*_μ_ being the mean intensity across the whole image and *I*_σ_ being the standard deviation of the intensity across the image. To ensure this resulted in a background level of zero, (*I*_μ_ + 2*I*_σ_) was subtracted from the images and all negative values were set to zero, leaving only positive values for the pixels with the strain field of the dislocations.

The cross-correlation image CC was then computed using the FFT of the set of weak-beam images:
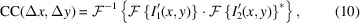

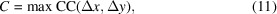
where 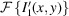 denotes the Fourier transform of the first image 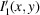 and 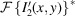 is the complex conjugate of the Fourier transform of the second image. The maximum value of CC(Δ*x*, Δ*y*) defines the *cross-correlation valueC* and the associated translational shift of (Δ*x*, Δ*y*).

## 3. Results

We first study the feasibility of dislocation identification by means of simulations, based only on a single image and with no additional information available, and then we compare with experimental data. In the latter case we will also consider the case of the slip plane being known (and hence the number of dislocation types reduced by a factor of 4). This information is readily available from 3D data, *i.e.* a stack of images acquired as a function of *z*.

### 3.1. Dislocation simulated image comparison

A series of weak-beam images of individual dislocations were simulated for diffraction on the [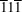] reflection. The images were simulated with 2θ = 17.953° and a sample orientation of
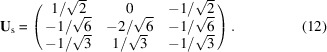


The test set included all combinations of the four slip planes, the six Burgers vectors allowed for each slip plane and line directions. These last were varied in 10° increments over the full dislocation loop. This results in a test set of 864 weak-beam images. These images we correlate with each other to quantify the likelihood of mistaken identities. Of the 864 configurations, the 24 that are very close to the invisibility criterion **G** · **b** = 0 (within 10°) have been excluded from the following discussion.

**Figure 4:**
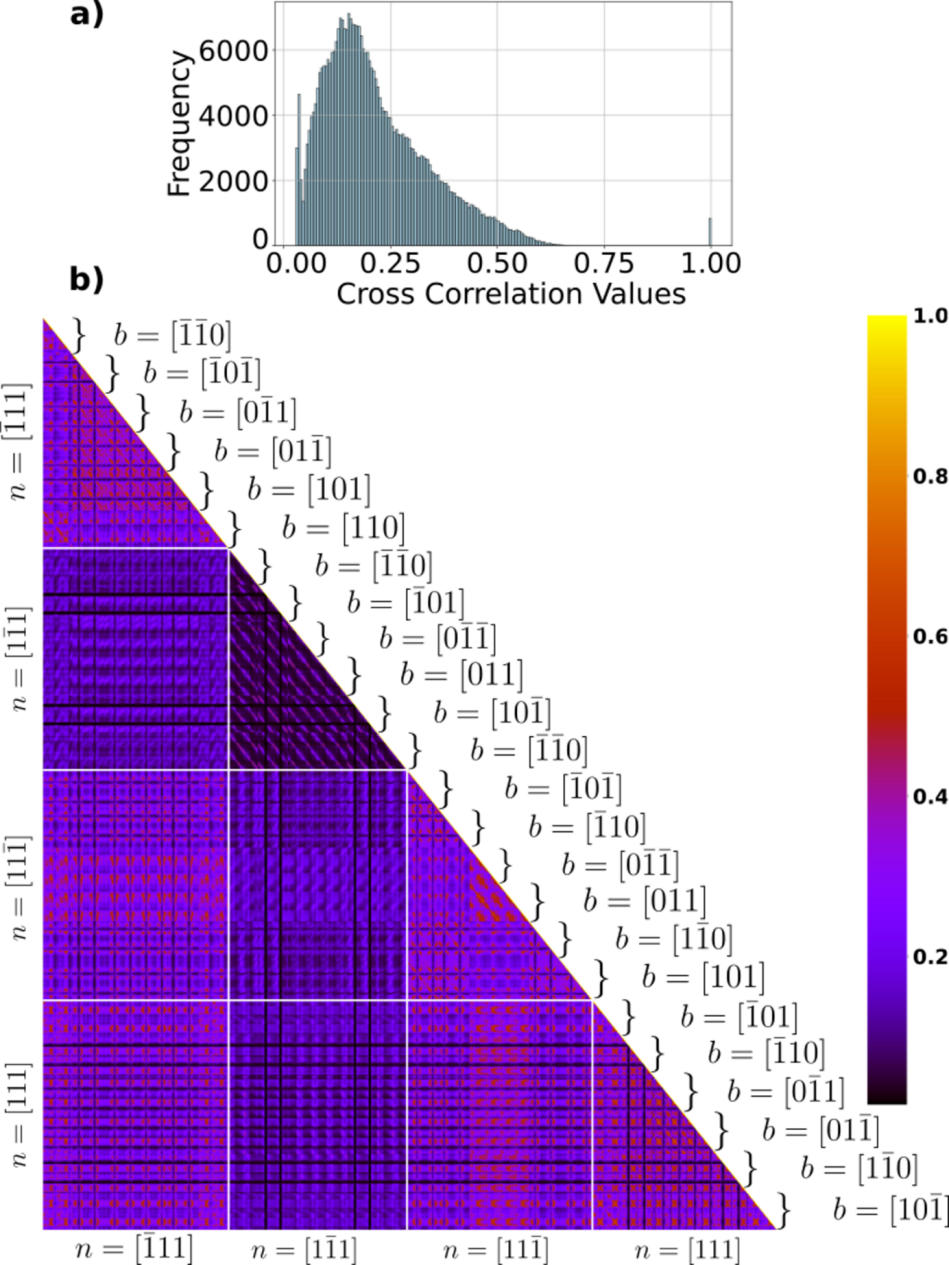
Covariance matrix of the cross-correlation values *C* for 840 simulated DFXM weak-beam images with [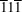] as scattering vector. Larger regions highlighted with white lines represent the four slip planes **n**. (Inset) Histogram of the cross-correlation values in the covariance matrix.

Fig. 4 ▸ displays the *covariance matrix* of the remaining 840 simulated images. Each pixel in the matrix represents the cross-correlation values between a set of two images, with the colour intensity indicating the degree of cross correlation. The matrix is structured in the following order (innermost to outermost loop):

(i) Line direction (10° increments)

(ii) Burgers vector (six for each slip plane)

(iii) Slip plane

This means that the first neighbour to a pixel differs by 10° in angle between the Burgers vector and dislocation line. Once the line direction has rotated 360°, the next group of 36 images has a different Burgers vector. The slip planes separated by white lines and their corresponding Burgers vectors are ordered as labelled in the horizontal direction.

The cross-correlation value intensity ranges from low (purple) to high (yellow), with the diagonal line showing perfect correlation between the same images. Note that all cross-correlation values related to non-identical images are below 0.69. Within each group of slip planes, it is clear that images with the same Burgers vector and slip-plane configuration exhibit similar correlation values. In some comparisons, *e.g.* between **n** = 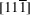 and **n** = 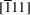, certain Burgers vectors consistently tend to give rise to higher correlation values (see the first column, second last row, as defined by the white lines in Fig. 4 ▸).

**Figure 5:**
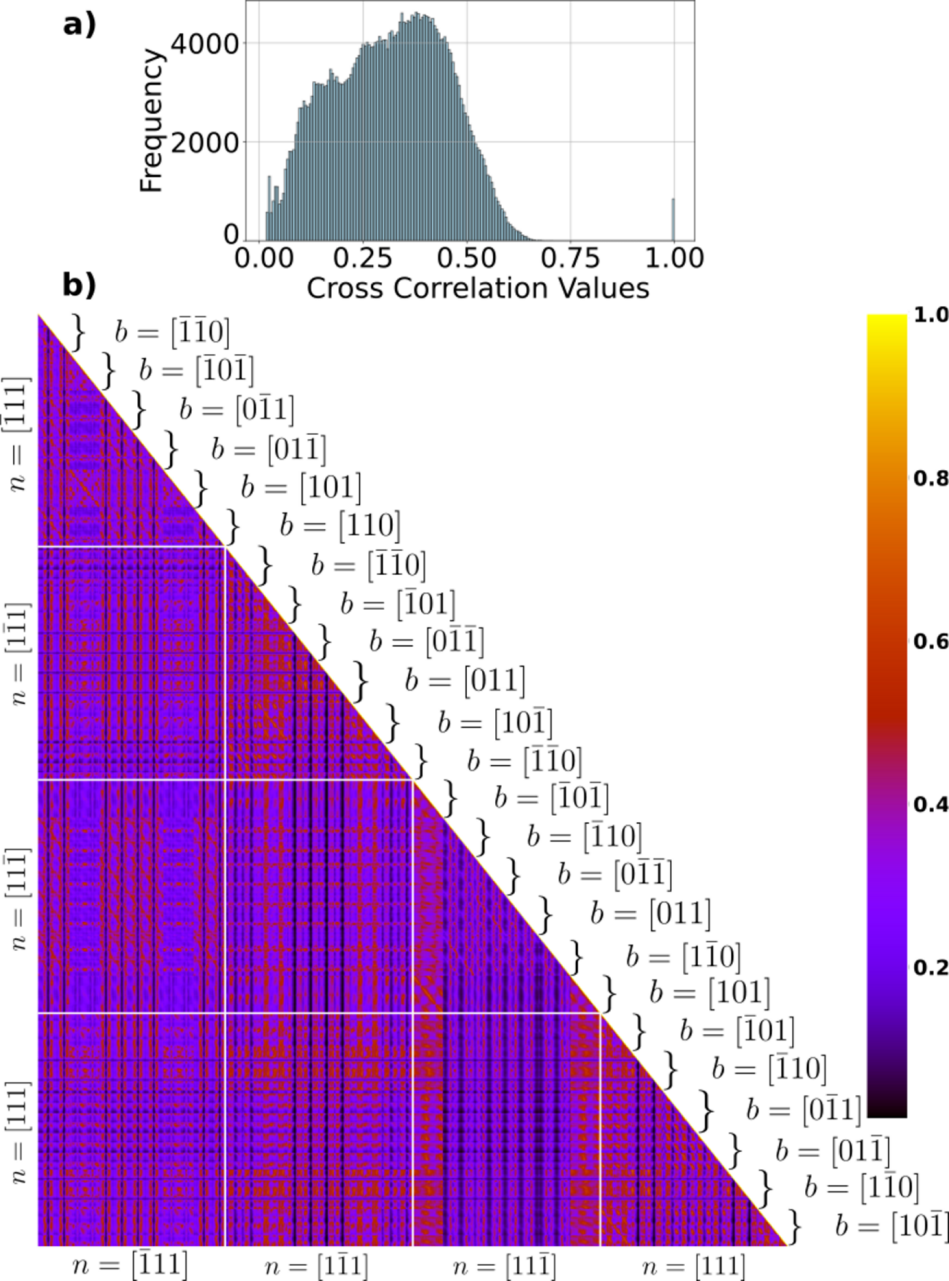
Covariance matrix of the cross-correlation values *C* for 846 simulated weak-beam images associated with a [020] scattering vector. Larger regions highlighted with white lines represent the four slip planes **n**. (Inset) Histogram of the cross-correlation values in the covariance matrix.

To test the significance of the chosen scattering vector, a similar covariance matrix was made from simulated weak-beam images for the [020] reflection. Here we used 2θ = 20.760° and a sample orientation of
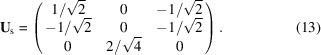
The resulting covariance matrix, based on 846 non-zero images, is shown in Fig. 5 ▸.

We observed that, by changing the scattering vector, the cross-correlation value distribution changed significantly – in this case the distribution shifted towards higher correlation values. However, the highest non-diagonal cross-correlation values only increased to 0.71. Here the covariance matrix revealed that specific Burgers vectors (groups of 36 columns or rows) show very high variation in the cross-correlation values. We also note that the variance between the different slip planes, regions highlighted by white lines in the figure, is much smaller than in Fig. 4 ▸, as all slip planes exhibit cross-correlation values with a higher mean.

### 3.2. Experimental identification and verification

The sample used in this study was mounted with the scattering vector for DFXM set to be [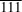]. During the experiment, a rocking curve was recorded across 11 layers, covering a total depth of 20 µm. Within this examined volume, several isolated dislocations were identified in a localized domain (Frankus *et al.*, 2025 ▸). By analysing the reconstructed 3D volume and considering the crystallographic orientation of the sample, we determined the slip plane on which the dislocations resided. Similarly, combining the knowledge of the crystallographic orientation with the information from the 3D construction, the line direction for each isolated dislocation was identified.

Next, we compared simulated images with the experimental images of the individual dislocations. However, when comparing experimental images with simulated images using the FFT cross-correlation method from equations (10) and (11), we found that some configurations exhibited high cross-correlation values despite being visibly poor matches upon inspection. To address this issue, a penalty was introduced in the comparison process by incorporating the mean square error (MSE) between the two images.

**Figure 6:**
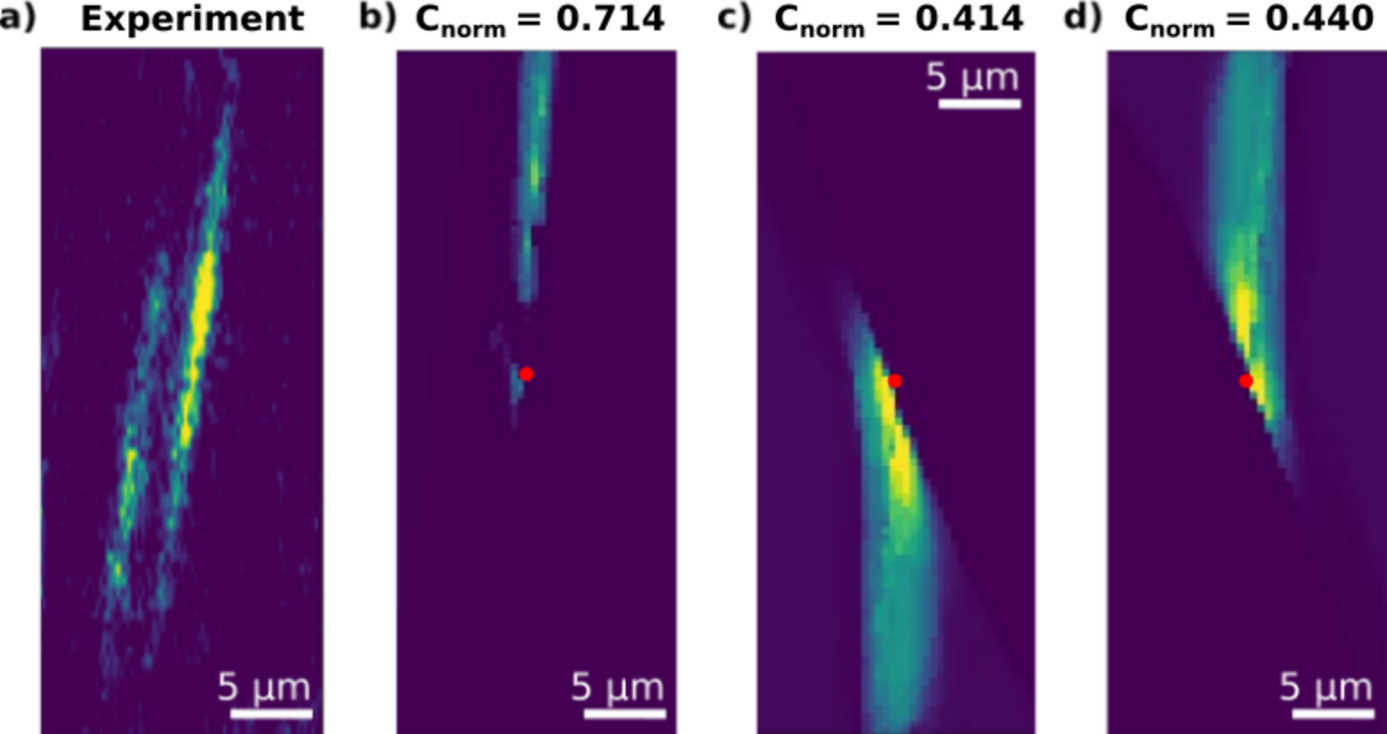
DFXM weak-beam images acquired at ϕ = 8 × 10^−4^°. (*a*) Experimental image. (*b*)–(*d*) Forward-modelled DFXM images with identical parameters to panel (*a*), representing a single dislocation at the centre (red dots) with Burgers vector (*b*) [110], (*c*) [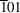] and (*d*) [011]. The normalized cross-correlation value, *C*_norm_, is indicated above each simulated image.

**Figure 7:**
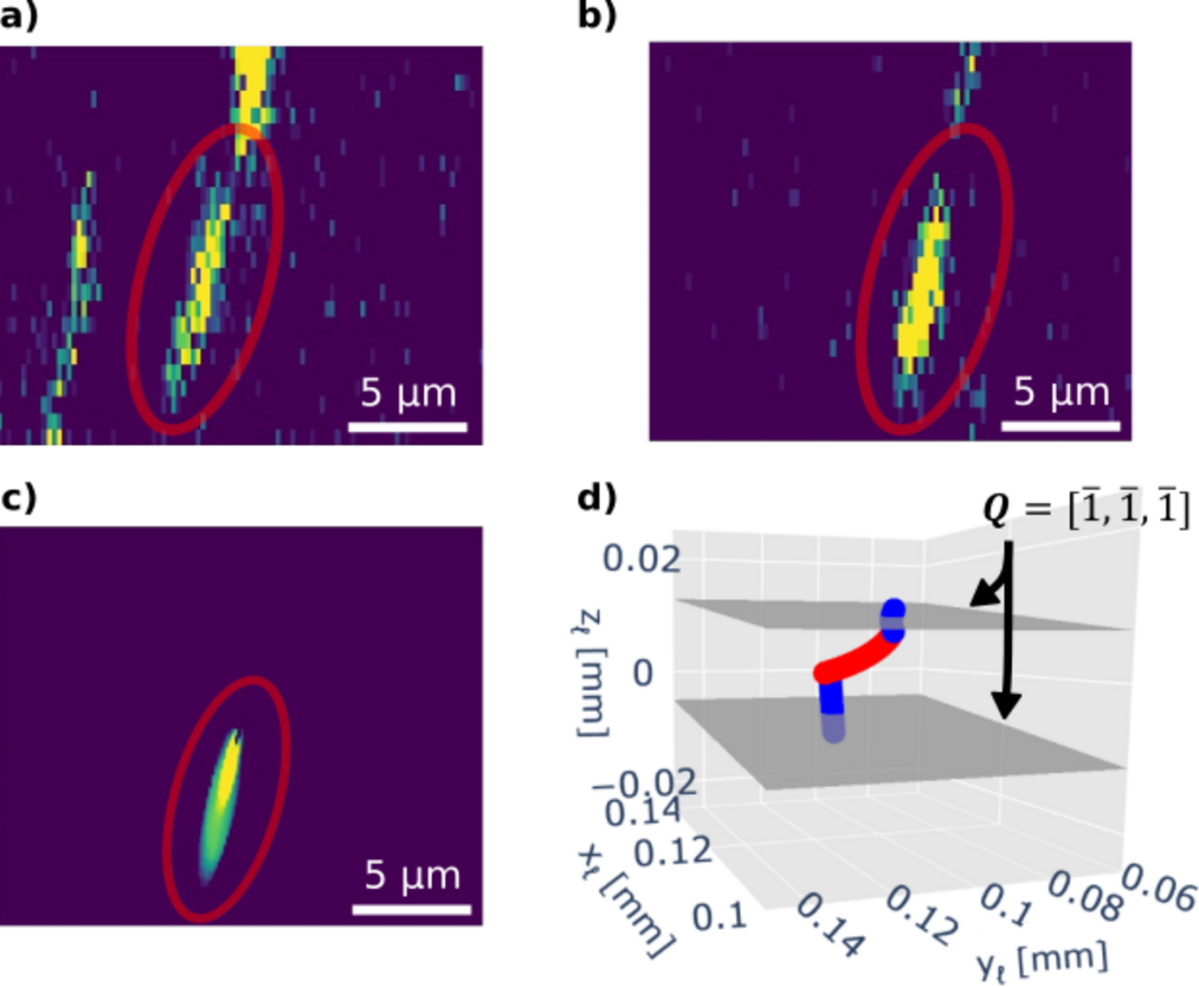
Double cross-slipping dislocation. (*a*) Weak-beam image of the lower blue segment in panel (*d*). (*b*) Weak-beam image of the upper blue segment in panel (*d*). (*c*) Corresponding forward-modelled image with the same parameters as the two segments of the dislocation. (*d*) Three-dimensional reconstruction of the double cross-slipping dislocation in laboratory coordinates. Diffraction planes are highlighted for images (*a*) and (*b*) with **Q** = 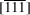.

When experimental images were compared with simulated images, there were cases where neighbouring dislocations were too close to the dislocation of interest to be properly centred in the cropped image, and this can be seen in Figs. 6(*a*) and 7(*a*). This was an issue for the MSE penalty, as it did not do any translation. Therefore, the translational shift from equation (10) (Δ*x*, Δ*y*) was incorporated into the MSE calculations, resulting in
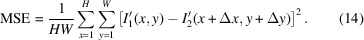
This was then used for the normalization of *C*,
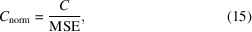
which can now be used for any cropped experimental image of an individual dislocation.

In Fig. 6 ▸(*a*) one individual dislocation, along with a nearby dislocation exhibiting a similar distortion field, is shown in a weak-beam image. This dislocation lies on the (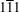) slip plane, restricting its possible Burgers vectors to [110], [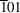] or [011]. In Figs. 6 ▸(*b*)–6 ▸(*d*), images of the three possible Burgers vectors have been forward modelled with GO, using the following **U**_s_: 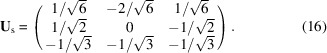
By visual comparison, as well as cross-correlation values, we conclude that the Burgers vector which exhibits a distortion field resembling the experimental image is [110], displayed in Fig. 6 ▸(*b*).

### 3.3. Example of use: a dislocation exhibiting double cross slip

With the experimental data from the sample described in Section 3.2, we observed a dislocation exhibiting double cross slip. The lower segment of the dislocation resided on the (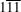) plane, while the cross-slipped portion lay on the (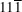) plane, before the upper segment returned to the (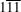) plane; see Figs. 7 ▸(*a*) and 7 ▸(*b*) for weak-beam images, and Fig. 7 ▸(*d*) for the reconstructed dislocation line in three dimensions. Note that the two diffraction planes shown in Fig. 7 ▸(*d*) are offset in the direction perpendicular to the slip plane. This configuration provided crucial information: the Burgers vector had to lie on both planes, and consequently it was determined to be [101]. Using the crystallographic and geometric data from the experimental images, we simulated the same dislocation (matching the line direction, Burgers vector, slip plane and sample orientation) [Fig. 7 ▸(*c*)] and achieved a near-perfect agreement between the simulated and experimental distortion fields through a visual comparison. For the purposes of this paper, this analysis serves as a way of establishing the ‘ground truth’.

Next we attempted to identify the dislocation type from a single weak-beam image without this analysis of the double cross-slip event. Specifically, the weak-beam image in Fig. 7 ▸(*b*) was compared with every possible dislocation type, in a similar manner to the covariance matrices in Figs. 4 ▸ and 5 ▸.

**Figure 8:**
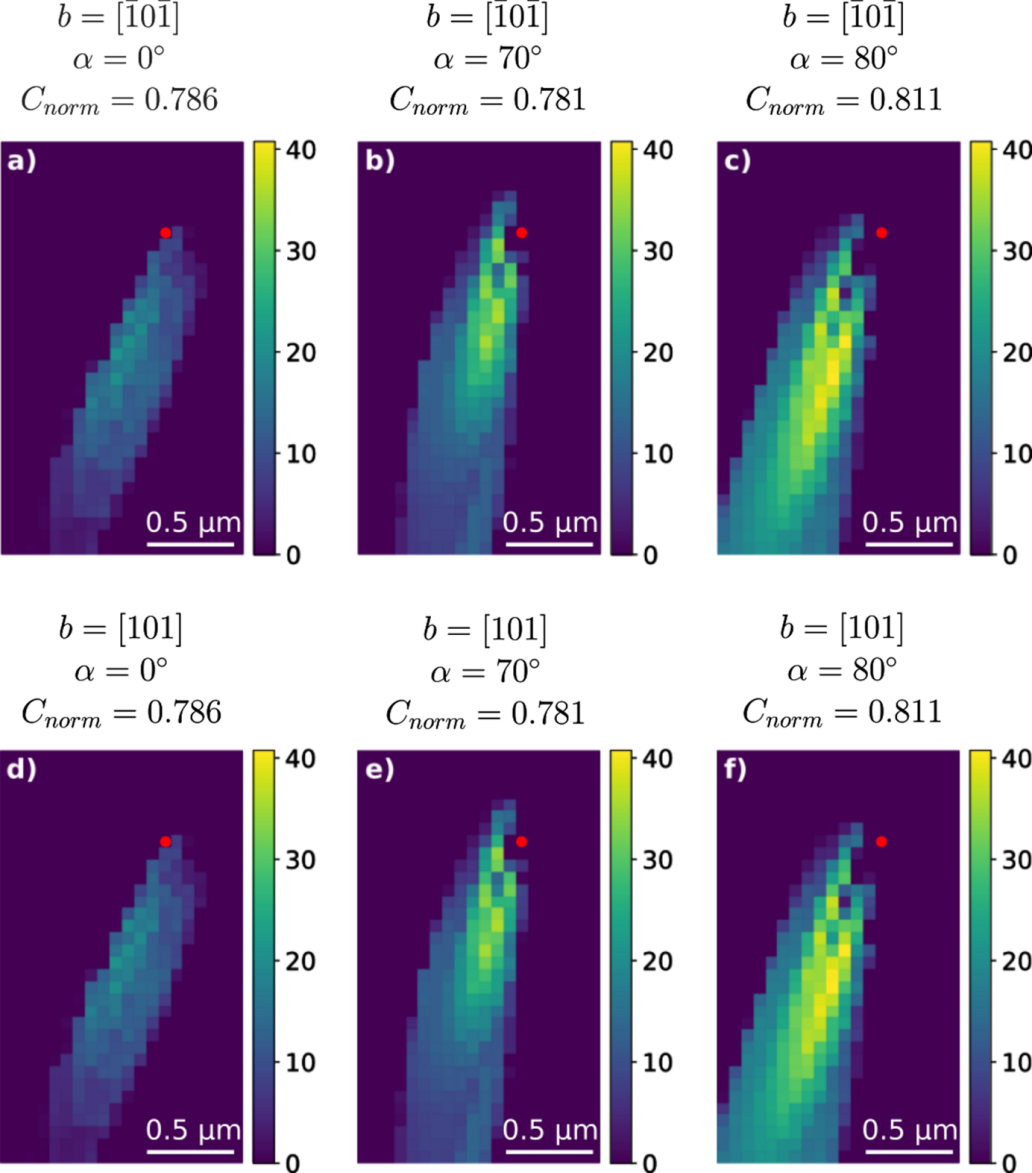
(*a*)–(*f*) Cropped weak-beam images (ϕ = 8 × 10^−4^°) of the six dis­lo­ca­tions with the highest *C*_norm_ values out of the 840 simulated ones. All dislocations lie on the (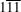) slip plane, and their Burgers vector **b** and line direction are displayed above each image. Here α represents the angle between the dislocation line direction and the Burgers vector **b**. The centres of the dislocations are marked with red dots.

The weak-beam image from Fig. 7 ▸(*b*) was preprocessed as described in Section 2.3. The same experimental parameters that were used for this image were used to generate 840 simulated weak-beam images of all possible individual dis­locations. The experimental image was cross correlated with the 840 simulated weak-beam images and the values are displayed in Fig. S2. The top six candidates from the normalized cross-correlation values are shown in Fig. 8 ▸. The next candidates had normalized cross-correlation values which were all more than 40% lower than the top six candidates.

Visual inspection confirms that the six dislocation types all exhibit a strain field which is similar to the experimental strain field. They all had the same Burgers vector [101], *i.e.* they were in accordance with the ground truth. The candidates displayed in Figs. 8 ▸(*a*) and 8 ▸(*d*) were of pure screw dislocation type, whereas the candidates displayed in Figs. 8 ▸(*b*), 8 ▸(*c*), 8 ▸(*e*) and 8 ▸(*f*) were of almost pure edge dislocation type.

When taking into account the information gained from the *z*-stacked images, the dislocation direction and slip planes are known. Here the slip plane for each candidate is correct and the Burgers vector is also correct. Moreover, we know from the *z*-stacked information that experimentally α = 72.72°, which excludes the candidates in Figs. 8 ▸(*a*) and 8 ▸(*d*) as they have α = 0°. This leaves us with the dislocations in Figs. 8 ▸(*b*), 8 ▸(*c*), 8 ▸(*e*) and 8 ▸(*f*) which have α = 70° and α = 80°. These four candidates have the same line direction within ±10° and the same Burgers vector apart from the sign: 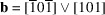.

## 4. Discussion

This work first of all validates the geometry and micro-mechanical model implemented in the geometrical optics code of Borgi *et al.* (2024 ▸) and demonstrates that, for isolated dislocations and in the optimal weak-field setting, DFXM images exhibit fields that are well described by forward projection of classical analytical expressions. This corroborates the potential of interfacing DFXM data to discrete dislocation dynamics simulations (Devincre *et al.*, 2011 ▸; Po *et al.*, 2014 ▸; Sills *et al.*, 2016 ▸; Pachaury *et al.*, 2022 ▸). In this connection it is relevant to note that the simulation of a single 510 × 170 pixel image which is used throughout this work with the GO code currently takes 0.33 s on a conventional laptop, while a 2000 × 2000 pixel image takes 182 s. Another potential application is the optimization of DFXM experiments prior to beamtime.

The work also demonstrates the identification of isolated dislocations with high fidelity, based on only one ϕ setting. This is a testimony to the displacement field giving rise to relatively large ‘blobs’ for the weak-beam setting applied. Unfortunately, spot overlap between these large blobs sets a lower limit on the dislocation density. Hence, it is relevant to consider acquiring data at more extreme ϕ values where only the core of the dislocation gives rise to diffraction. With decreasing spot sizes it becomes increasingly relevant to include all images from a rocking scan in the identification process. More generally, identification may be informed additionally by scanning in the χ and 2θ directions as well, and by probing more than one diffraction vector. Such procedures are outside the scope of this paper.

Next, we argue that dislocation identification of the kind studied here may benefit from the use of machine learning tools. The current GO code is able to provide training data sets comprising tens of thousands of images. As an inspiration, we note that machine learning has been powerful for classification tasks in orientation mapping methods such as EBSD, enabling an improvement in resolution and/or a reduction in exposure times by a factor of 5–10 (Ding *et al.*, 2021 ▸). The latter is very relevant for dislocation dynamics studies.

Comparing Figs. 4 ▸ and 5 ▸, it is clear that the choice of reflection plays a role in how easy it is to identify a dislocation from a single weak-beam image. This is prominent for dis­locations that lie in the diffraction plane, *e.g.* the (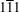) slip plane with a [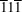] reflection. Here we see the lowest cross-correlation values compared with all other slip planes. This is because the dislocations on the slip plane lie exactly in the illuminated plane, making them very distinguishable from each other by just a simple 10° rotation of the line direction. However, the gauge volumes of each weak-beam image in this DFXM setup are often very anisotropic, with the width and length of the illuminated volume of the order of 800–1600 times larger than the height of the illuminated volume. This results in a significantly smaller chance of finding dislocations that lie exactly in the diffraction plane of a single weak-beam image. If one were to acquire a *z* stack of weak-beam images, obtaining a full 3D volume, the chance of finding dislocations within the diffraction plane increases.

For these simulated images, only a single weak-beam image of each dislocation configuration was simulated. Therefore we now take a closer look at dislocations that lie on other slip planes than (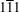). Here there are still variations between the other slip planes when comparing the two reflections. As an example, looking at dislocations that lie on the (111) slip plane, they have lower cross-correlation values with the [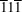] reflection when comparing them not just with dislocations that lie on the same plane but also with those in other slip planes. This suggests that careful consideration of the chosen reflection can be essential during experimental planning.

In relation to the six candidate dislocation types exhibited in Fig. 8 ▸, we note that the peak intensities for the two screw dislocations, represented by panels (*a*) and (*d*), are half those of the other four dislocations. This discrepancy suggests that these two configurations can be excluded as possible matches, narrowing the candidates to four without the use of any further information.

Straining the experimental sample to reveal in which direction it moves could potentially reveal the sign of the Burgers vector, reducing the set to just two candidates, differing by only ±10° in α. For many applications this accuracy is sufficient.

Finally, we discuss this work in the context of the recent study by Pal *et al.* (2025 ▸). Using the same geometrical optics framework, they extend the ‘invisibility criterion’ formalism known from TEM to DFXM. Next, they explore how the asymmetry of rocking tilt scans at different rolling tilts could be used develop a different method to characterize the Burgers vector. It would be interesting to compare results from actual measurements.

## 5. Conclusions

The comparisons of the forward simulations of DFXM images and the experimental images in Figs. 6 ▸ and 7 ▸ demonstrate that the geometrical optics tool can accurately model DFXM weak-beam images that closely resemble experimental results. This establishes the simulation tool as a reliable addition to DFXM data analysis for dislocation studies.

The cross correlation of weak-beam images across all possible configurations of an individual dislocation shows that, within a certain confidence level, no images are too similar to prevent successful identification of dislocation type using only a single weak-beam image. Scanning the sample in *z* substantially reduces the number of candidate dislocation types by identifying the slip plane and line direction. We note that additional information is readily available from scanning the sample in χ and/or ϕ and *e.g.* relying on centre-of-mass maps, although this is outside the scope of the present work. In total, this should enable robust dislocation type identification with a high confidence.

This work paves the way for utilizing supervised machine learning with DFXM data by making use of the GO simulations as training sets. Additionally, it offers opportunities to incorporate dislocation dynamics modelling, such as CDD or DDD, to track the evolution of dislocation structures during straining. This approach could enable direct comparisons with experimental 3D movies of dislocation evolution within bulk samples.

## Supplementary Material

Additional figures. DOI: 10.1107/S1600576725002614/nb5396sup1.pdf
