# Supplementary material for: Individual dislocation identification in dark-field X-ray microscopy
Source: J Appl Crystallogr. 2025 May 2;58(Pt 3):813–21. doi: 10.1107/S1600576725002614 (PMC12135992; doi:10.1107/S1600576725002614)
Supplement: Supplementary file 1 [file j-58-00813-sup1.pdf]

JOURNAL OF APPLIED CRYSTALLOGRAPHY

Volume 58 (2025)

Supporting information for article:

Individual dislocation identification in dark-field X-ray microscopy

Sina Borgi, Grethe Winther and Henning Friis Poulsen

Supplementary Materials for
Individual Dislocation Identification in Dark-Field X-ray Microscopy

S. Borgi,[a] G. Winther,[b] and H. F. Poulsen[a*]

[a] *Department of Physics, Technical University of Denmark, Lyngby, Denmark*
[b] *Department of Civil and Mechanical Engineering, Technical University of Denmark, Lyngby, Denmark*

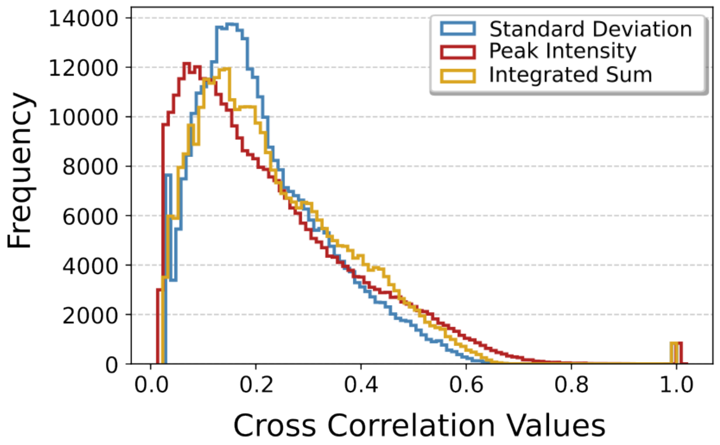

Figure S1: Histograms of cross correlation values for three different image normalization techniques. (blue) Z-score normalization: The input image is standardized by subtracting the mean and 2σ and then divided by σ. (red) Max intensity normalization: Each image is divided by its maximum intensity value. (yellow) Total sum normalization: Each image is divided by its total sum, resulting in a normalized image where the sum of pixel values equals to 1. The histograms are based on the dataset used for Fig. 4 with $\mathbf{Q} = [\bar{1}, \bar{1}, \bar{1}]$.

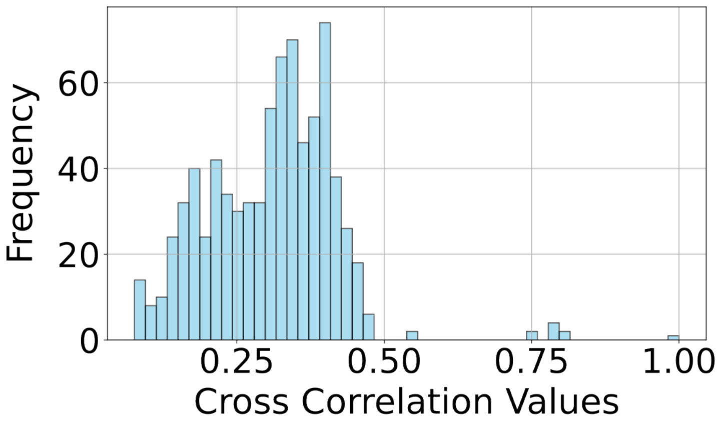

Figure S2: Histogram of cross correlation values for the experimental weak beam image in Fig. 7b with 840 simulated weak beam images from the same reflection, each displaying a dislocation with unique parameters (Burgers vector, slip plane and line direction). Excluding the experimental image itself (cross correlation value of 1.0), the 6 weak beam images with the highest cross correlation values are plotted in Fig. 8.
